# Mast Cells and Substance P: Neuroinflammatory Loops at the Molecular and Translational Clinical Levels

**DOI:** 10.3390/biom16040539

**Published:** 2026-04-04

**Authors:** Ernesto Aitella, Marilena Bruno, Gianluca Azzellino, Massimo De Martinis, Lia Ginaldi, Ciro Romano

**Affiliations:** 1 Department of Life, Health and Environmental Sciences, University of L’Aquila, 67100 L’Aquila, Italy; ernesto.aitella@graduate.univaq.it (E.A.); marilena.bruno@graduate.univaq.it (M.B.); gianluca.azzellino@graduate.univaq.it (G.A.); massimomariamarcello.demartinis@univaq.it (M.D.M.); lia.ginaldi@univaq.it (L.G.); 2 Allergy and Clinical Immunology Unit, Center for the Diagnosis and Treatment of Osteoporosis, Azienda Unità Sanitaria Locale 04 Teramo, 64100 Teramo, Italy; 3 Complex Operational Unit, Adriatic District Area, Azienda Unità Sanitaria Locale 04 Teramo, 64100 Teramo, Italy; 4 Long-Term Care Unit, “Maria SS. dello Splendore” Hospital, Azienda Unità Sanitaria Locale 04 Teramo, 64021 Teramo, Italy; 5 UniCamillus-Saint Camillus International University of Health Sciences, 00131 Rome, Italy; 6 Clinical Immunology Outpatient Clinic, Division of Internal Medicine, Department of Advanced Medical and Surgical Sciences, “Luigi Vanvitelli” University of Campania, 80131 Naples, Italy

**Keywords:** mast cells, substance P, neurogenic inflammation

## Abstract

Mast cells, characterized by a broad repertoire of surface receptors, are increasingly recognized for activation pathways extending beyond the classical IgE/FcεRI axis, particularly in the context of neurogenic inflammation. Substance P (SP), a neuropeptide of the tachykinin family, is a potent activator of mast cells, inducing the release of histamine, cytokines, and other inflammatory mediators. Through complex bidirectional communication, mast cells and SP play a pivotal role in neuro–immune interactions. This narrative review provides an updated overview of mast cell–SP crosstalk, with a focus on underlying molecular mechanisms, receptor-mediated signaling pathways, and their contribution to pathophysiological processes. In addition, we aim to reinterpret established clinical models within the spectrum of pseudoallergic conditions and to explore innovative, etiology-driven therapeutic strategies. Finally, we discuss future perspectives and highlight the need for robust translational models to support clinical and pharmacological research.

## 1. Introduction

Traditionally, mast cells have been primarily considered in the context of allergic diseases, in which the canonical activation pathway is represented by IgE-mediated cross-linking of FcεRI-bound antibodies [1]. This mechanism provides the “textbook” explanation for immediate hypersensitivity reactions and type 2 inflammation, with downstream consequences including itch, bronchospasm, mucus hypersecretion, and tissue edema. However, it is increasingly evident that mast cell biology extends beyond IgE [2]. Over the last decade, multiple IgE-independent pathways have been characterized and linked to clinically relevant phenomena ranging from pseudoallergic drug reactions to neurogenic inflammatory cascades. Among these, the Mas-related G protein-coupled receptor X2 (MRGPRX2) has emerged as a key mast cell receptor at the crossroads of innate immunity, neuropeptide signaling, and drug hypersensitivity. Unlike FcεRI, MRGPRX2 activation does not require prior sensitization and can be triggered by a broad spectrum of ligands, including endogenous neuropeptides, host defense peptides, and several pharmacological agents [3]. This feature positions MRGPRX2 as a plausible mechanistic substrate for inflammatory phenomena characterized by rapid onset, unpredictable recurrence, and poor alignment with traditional IgE biomarkers.

Substance P (SP), an 11–amino acid tachykinin encoded by the *TAC1* gene, represents a well-established example of the neuroimmune role of neuropeptides. Historically recognized as a neurotransmitter involved in pain transmission, SP is now regarded as a multifunctional mediator capable of coordinating neuronal, immune, and vascular responses [4]. Importantly, SP is not produced exclusively by neurons: it can also be synthesized and released by several non-neuronal immune and structural cells, including mast cells themselves, T lymphocytes, monocytes/macrophages, dendritic cells, epithelial cells, and endothelial cells. In tissues, SP is rapidly degraded by enzymes such as neprilysin.

The best-characterized receptor for SP is the neurokinin-1 receptor (NK1R), which is widely expressed in neuronal and non-neuronal cells [5]. NK1R signaling activates canonical G protein-coupled receptor pathways and can propagate both acute effects (calcium mobilization and vascular changes) and longer-term transcriptional programs (NF-κB-driven cytokine expression). Receptor internalization and β-arrestin recruitment add further layers of temporal regulation, supporting sustained intracellular signaling even after ligand exposure. Nevertheless, SP-induced mast cell activation is not limited to NK1R but also involves MRGPRX2. The identification of MRGPRX2 as an SP-responsive mast cell receptor provides a mechanistic bridge between neurogenic inflammation and pseudoallergic responses, supporting a model in which SP can activate mast cells rapidly and independently of sensitization.

A distinctive feature of mast cell–SP interactions is their ability to generate self-reinforcing loops. SP induces mast cell degranulation, with the release of histamine and proteases that promote vasodilation, increased permeability, and immune cell recruitment. Mast cell tryptase can activate protease-activated receptor-2 (PAR-2) on sensory nerve terminals, facilitating further SP release and enhancing nociceptor excitability [6]. This feed-forward circuit provides an elegant explanation for the amplification of symptoms such as pain, itch, and localized edema, particularly in conditions characterized by dense tissue innervation and abundant mast cells. In this sense, neurogenic inflammation can be conceptualized not as a unidirectional neuron–immune event, but rather as a bidirectional circuit in which mast cells act both as effectors and as modulators of neuronal activity.

Beyond peripheral tissues, growing interest has focused on neurogenic inflammation within the central nervous system (CNS). Mast cells have been identified in the meninges, perivascular spaces, and neuroendocrine regions such as the hypothalamus [7]. In these sites, mast cells interact with neurons, astrocytes, microglia, and the vascular endothelium of the blood–brain barrier (BBB). SP signaling may contribute to changes in BBB permeability and promote neuroinflammation through the release of mast cell mediators.

This narrative review was conducted through a structured literature search aimed at identifying the most relevant experimental and clinical studies on the mast cell–SP axis. The primary databases consulted were PubMed/MEDLINE, Scopus, and Web of Science. The search covered the period from January 2000 to January 2026, with additional landmark studies included for historical and mechanistic context. The main search terms included combinations of “mast cells”, “substance P”, “MRGPRX2”, “neurokinin-1 receptor”, “neurogenic inflammation”, “pseudoallergic reactions”, and “neuroimmune interaction”.

Studies were selected based on their relevance to receptor signaling mechanisms, mast cell activation pathways, neuroimmune crosstalk, and translational or clinical implications. Both preclinical and human studies were considered. Particular attention was given to experimental models allowing mechanistic interpretation and to clinical investigations providing evidence of disease association or therapeutic modulation.

Given the heterogeneity of available data, this review does not aim to provide a systematic quantitative synthesis but rather a concept-driven integration of molecular, cellular, and clinical evidence to frame the mast cell–SP axis as a modular neuroimmune entity linking mechanistic pathways to complex clinical phenotypes.

## 2. Mast Cells: Phenotypes and Mediator Repertoire

Neurogenic inflammation is a complex biological process resulting from the dynamic interaction between the nervous and immune systems and is characterized by the release of neuropeptides from sensory nerve endings, leading to vascular, immune, and neuronal responses. Among the mediators involved, SP and mast cells play a central and mutually reinforcing role [8]. Mast cells are tissue-resident immune cells that originate from hematopoietic stem cells in the yolk sac and bone marrow under the influence of stem cell factor and subsequently migrate to peripheral tissues, where they complete their maturation [9]. Once differentiated, mast cells localize preferentially at neurovascular interfaces, including the skin, respiratory and gastrointestinal mucosa, meninges, and perivascular regions of the central and peripheral nervous systems [10]. Their strategic positioning allows them to function as sentinels at sites where environmental, immune, and neuronal signals converge [11].

Upon activation, mast cells exert profound biological effects through both rapid degranulation and de novo synthesis of mediators [12,13]. These include biogenic amines such as histamine and serotonin, proteases including tryptase and chymase, lipid-derived mediators such as prostaglandins and leukotrienes, and a broad array of cytokines, chemokines, and growth factors [9,14,15]. Through these mediators, mast cells regulate innate and adaptive immune responses, modulate inflammation, influence vascular permeability, and contribute to tissue repair, fibrosis, angiogenesis, and tumor progression [16,17,18,19]. Human mast cells are classically divided into two major subtypes based on their protease content: connective tissue mast cells (MCTC), which are abundant in the skin and perivascular tissues, containing both tryptase and chymase, and mucosal mast cells (MCT), which predominate in the respiratory and gastrointestinal mucosa, containing tryptase only [20,21]. Importantly, MCTC display a receptor profile that renders them particularly responsive to neuropeptides, underscoring their role in neurogenic inflammation [22].

Mast cells exhibit substantial phenotypic heterogeneity that is influenced by the local cytokine milieu and tissue microenvironment [23], as also supported by transcriptomic analyses [24,25,26]. In addition, several mast cell markers are expressed independently of maturation or activation status. For example, CD117 is present across mast cell populations regardless of activation state, while the expression of other surface antigens may vary according to tissue environment, maturation stage, and disease context [27,28]. This variability reflects the complex interplay between mast cells and microenvironmental signals across different tissues [29]. Consequently, commonly used immunohistochemical markers such as tryptase, chymase, and CD117 primarily identify mast cell presence rather than specific activation states. Notably, they cannot be considered definitive indicators of mast cell activation and should therefore be interpreted cautiously [30], highlighting the need for biomarkers capable of distinguishing mast cell activation from mast cell burden and underscoring current diagnostic limitations [31].

### 2.1. Mast Cell Activation Pathways: IgE-Mediated and Non-IgE Mechanisms

Mast cells are traditionally recognized as key effectors of T-helper-2-dominated immune responses and allergic inflammation [32,33]. In this context, activation occurs primarily through the high-affinity immunoglobulin E receptor FcεRI. Cross-linking of FcεRI-bound IgE by specific allergens initiates intracellular signaling cascades involving phospholipase C activation, inositol triphosphate generation, intracellular calcium mobilization, and engagement of mitogen-activated protein kinase pathways. These events culminate in immediate degranulation with release of preformed mediators, followed by delayed synthesis of lipid mediators and cytokines such as interleukin-4, interleukin-5, interleukin-9, and interleukin-13 [34]. Histamine and interleukin-4 play key roles in allergic inflammatory disorders such as urticaria, atopic and allergic contact dermatitis, by inducing pruritus through activation of sensory nerve fibers and by promoting type 2 immune responses characterized by increased vascular permeability and altered smooth muscle tone [35,36,37].

In addition to IgE-dependent mechanisms, mast cells can be activated through IgE-independent pathways that are highly relevant to neurogenic inflammation [38,39]. A key receptor mediating this process is MRGPRX2, which is preferentially expressed on MCTC [40,41]. MRGPRX2 belongs to the family of Mas-related G protein-coupled receptors, characterized by seven transmembrane α-helices, and was initially identified in murine mast cells as Mrgprb2, with subsequent characterization of the human ortholog. Unlike FcεRI-mediated activation, MRGPRX2 signaling does not require prior sensitization and is rapidly triggered by a wide range of endogenous and exogenous ligands, including neuropeptides, host defense peptides, and several clinically used drugs [42].

SP represents one of the most potent endogenous activators of mast cells through MRGPRX2 [43]. Binding of SP to the extracellular domain of MRGPRX2 induces conformational changes that enable G protein coupling and activation of downstream signaling pathways [44,45], with a critical requirement for Ca^2+^ influx and PI3K activation, and additional modulatory contributions from ERK1/2 pathways [46]. This results in rapid mast cell degranulation and the release of histamine, tryptase, and chemokines, which promote vasodilation, increased vascular permeability, and recruitment of innate immune cells such as neutrophils, monocytes, and macrophages [47,48]. Mast cell-derived tryptase further activates PAR-2 on sensory afferent neurons, stimulating additional release of SP and establishing a feed-forward loop that amplifies local inflammatory responses [49,50]. This bidirectional communication between mast cells and sensory neurons is a defining feature of neurogenic inflammation and contributes to peripheral nerve sensitization, pain, and itch [51,52,53].

### 2.2. Receptor Hierarchy in Human Mast Cells: NK1R Versus MRGPRX2

Although SP can signal through both NK1R and MRGPRX2, their relative contribution to mast cell activation appears to differ substantially in human systems. Several studies have reported limited or variable expression of NK1R in human skin mast cells, whereas MRGPRX2 is consistently and highly expressed, particularly in connective tissue mast cells. This distribution suggests that rapid SP-induced degranulation in human tissues is predominantly mediated by MRGPRX2 rather than NK1R [19].

Functional data further support this hierarchy. MRGPRX2 activation induces immediate calcium influx and rapid exocytosis of preformed mediators, a kinetic profile that closely matches the early vascular and neurogenic responses observed in vivo [46]. In contrast, NK1R signaling in mast cells appears to be more closely associated with transcriptional regulation, cytokine production, and sustained inflammatory responses, although its functional relevance in primary human mast cells remains less clearly defined [42,54].

Important interspecies differences must also be considered. The murine ortholog Mrgprb2 shares functional similarities with human MRGPRX2 but differs in ligand selectivity and signaling properties, limiting direct translational extrapolation [55]. This distinction is critical when interpreting data derived from rodent models of neurogenic inflammation and pseudoallergic reactions.

Current evidence supports a model in which MRGPRX2 represents the dominant receptor for SP-induced rapid mast cell activation in humans, whereas NK1R may play a complementary or context-dependent role, particularly in regulating late-phase inflammatory programs. In fact, MRGPRX2 and NK1 receptors activate partially overlapping but functionally distinct pathways. MRGPRX2 primarily triggers rapid G-protein-dependent signaling leading to calcium influx and immediate mast cell degranulation. In contrast, NK1 receptor activation more commonly engages β-arrestin recruitment, receptor internalization, and sustained MAPK and NF-κB signaling, contributing predominantly to cytokine production and longer-term inflammatory responses [4,53,56].

Moreover, NK1R desensitization is well-documented: multiple studies demonstrate that SP induces rapid NK1R desensitization through phosphorylation and β-arrestin recruitment [57,58,59]. This represents classical G protein-coupled receptor desensitization mechanisms.

For MRGPRX2, the evidence appears more nuanced: while SP has been shown to induce receptor desensitization through β-arrestin recruitment [56], other studies emphasize the rapid secretory responses and distinctive trafficking dynamics associated with MRGPRX2 activation [60,61]. Interestingly, a potential relationship exists between MRGPRX2 desensitization and rapid mast cell degranulation. In ex vivo human skin mast cells, maximal responses were observed after 1 min following MRGPRX2 stimulation, whereas FcεRI-triggered activation reached its peak only after 8 min [62].

## 3. Substance P Biology and Receptor Signaling

SP is an amphiphilic neuropeptide composed of 11 amino acids and belongs to the tachykinin family. It is generated from the precursor preprotachykinin A, encoded by the *TAC1* gene located on chromosome 7. Alternative splicing of the *TAC1* gene gives rise to multiple mRNA variants, enabling the production not only of SP but also of other tachykinins, including neurokinin A (NKA), neurokinin B (NKB), neuropeptide K, and neuropeptide γ [63,64,65]. Although these peptides share structural similarities, they display preferential receptor selectivity: SP primarily activates NK1R, NKA preferentially binds neurokinin-2 receptor (NK2R), and NKB mainly signals through neurokinin-3 receptor (NK3R) [66]. Among these tachykinins, SP appears to play the most prominent role in mast cell activation and neuroimmune signaling [67].

In humans, *TAC1* expression is particularly high in transient receptor potential vanilloid 1–positive nociceptive neurons, highlighting the close association between SP and pain signaling pathways [18,48,68].

Although SP is classically regarded as a neurotransmitter, it is now well established that it is also produced and released by a wide range of non-neuronal cells [69]. These include mast cells, T lymphocytes, monocytes, macrophages, dendritic cells, endothelial cells, epithelial cells, and eosinophils [15,18,70,71]. This broad cellular distribution underscores the role of SP as a neuroimmune mediator capable of coordinating responses across multiple biological systems [72]. SP is highly conserved among mammalian species and exerts its biological effects exclusively through receptor-mediated mechanisms, as it is unable to cross cellular membranes [65,73]. The positively charged N-terminal region of the peptide is critical for receptor binding, whereas the hydrophobic C-terminal region confers amphiphilic properties [48,74,75].

In tissues, SP has a short half-life ranging from seconds to minutes and is rapidly degraded by cell-surface metalloproteases such as neprilysin [76]. Its biological actions are mediated primarily through neurokinin receptors and MRGPRX2 [77]. Neurokinin receptors are G protein–coupled receptors widely expressed on neurons, immune cells, endothelial cells, smooth muscle cells, fibroblasts, and mast cells. Among the three known subtypes, the NK1R exhibits the highest affinity for SP and exists in both full-length and truncated isoforms with distinct tissue distributions and signaling capacities [5]. In the nervous system, NK1R receptor expression is upregulated in dorsal horn neurons during inflammation, facilitating enhanced nociceptive transmission [48,70,78,79,80].

Engagement of SP with NK1R or MRGPRX2 activates intracellular signaling pathways, including the mitogen-activated protein kinase, phosphoinositide 3-kinase–Akt, and nuclear factor kappa B pathways [20,81,82]. Binding of SP to NK1R induces receptor phosphorylation by G protein-coupled receptor kinases and subsequent recruitment of β-arrestin, leading to receptor internalization and desensitization while also supporting sustained intracellular signaling [48,83]. Activation of phospholipase C results in inositol triphosphate–mediated calcium release and diacylglycerol-dependent activation of protein kinase C, whereas stimulation of adenylyl cyclase generates cyclic adenosine monophosphate and activates protein kinase A [68]. These signaling cascades converge on transcription factors such as nuclear factor kappa B and peroxisome proliferator-activated receptors, promoting the expression of pro-inflammatory cytokines, chemokines, and adhesion molecules [15,53,84,85].

**Figure 1:**
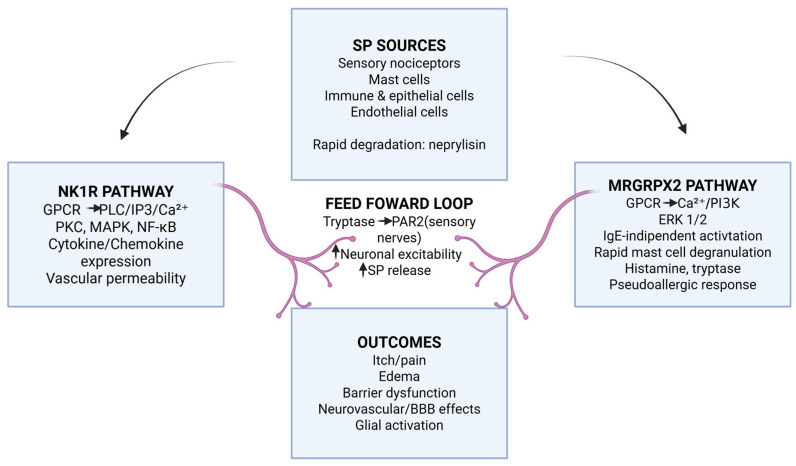
Substance P-mast cell neuroimmune axis. SP released from sensory nociceptors and non-neuronal cells engages two parallel receptor pathways on mast cells. NK1R activation promotes intracellular signaling cascades leading to cytokine and chemokine production and vascular effects, whereas MRGPRX2 activation induces rapid, IgE-independent mast cell degranulation with release of histamine and proteases. Mast cell tryptase activates protease-activated receptor-2 (PAR-2) on sensory nerve endings, generating feed-forward loops that amplify SP release, neuronal excitability, barrier dysfunction, and neuroinflammatory signaling, including effects at the neurovascular unit and blood–brain barrier. SP, substance P; NK1R, neurokinin-1 receptor; MRGPRX2, Mas-related G protein-coupled receptor X2; GPCR, G protein-coupled receptor; PLC, phospholipase C; IP3, inositol trisphosphate; PKC, protein kinase C; MAPK, mitogen-activated protein kinase; NF-κB, nuclear factor kappa B; PI3K, phosphatidylinositol 3-kinase; ERK1/2, extracellular signal-regulated kinases 1/2; PAR-2, protease-activated receptor 2; BBB, blood–brain barrier; IgE, immunoglobulin E. Created with BioRender, Ginaldi L. (2026) https://BioRender.com/tfn7he8 (accessed on 29 January 2026).

The downstream effects of SP signaling include enhanced neuronal excitability, modulation of synaptic transmission, and facilitation of immune cell recruitment to sites of inflammation (Figure 1). In the spinal cord, SP contributes to central sensitization by enhancing the function of α-amino-3-hydroxy-5-methyl-4-isoxazolepropionic acid (AMPA) and N-methyl-D-aspartate (NMDA) receptors and sustaining inflammation-induced hyperexcitability [70,86,87]. This process transforms non-nociceptive neurons into nociceptive neurons and perpetuates chronic pain states [20,88]. Peripheral and central sensitization are further supported by the increased production of arachidonic acid metabolites, including prostaglandins and lipoxygenase products, which act synergistically with SP to maintain inflammatory signaling [84,89].

SP also promotes vascular effects, including endothelial activation, vasodilation, increased vascular permeability, and edema formation by inducing the expression of endothelial leukocyte adhesion molecules and stimulating nitric oxide and vascular endothelial growth factor release [15,90]. Through these mechanisms, SP facilitates leukocyte extravasation and amplifies inflammatory responses [18,91]. Functionally, SP plays a fundamental role in nociception by increasing postsynaptic sensitivity to glutamate, enhancing neuroactive mediator release at nociceptor terminals, and stimulating trigeminal and spinal pain pathways. Beyond pain transmission, SP influences emotional processing, stress responses, and mood regulation, linking neurogenic inflammation to anxiety and depressive disorders [20,48,84,92,93].

## 4. Mast Cell–Substance P Axis: Experimental Models

The experimental evidence of the mast cell-SP axis derives from different model systems that vary in their capacity to reproduce physiological mast cell responses, including immortalized mast cell lines, primary human mast cells, and animal models of neurogenic inflammation (Table 1). These cellular models differ in origin and physiological relevance [94]. Primary human mast cells can be isolated from tissues such as skin or lung through enzymatic digestion followed by density-gradient separation. Alternatively, mast cells can be generated in vitro from hematopoietic progenitors obtained from peripheral blood or umbilical cord blood and differentiated under cytokine stimulation. In addition, immortalized mast cell lines such as LAD2 and HMC-1 are widely used for mechanistic studies because they provide reproducible systems for investigating intracellular signaling pathways. These complementary models form the experimental basis for the evidence summarized in the following sections.

### 4.1. Mast Cell Lines

Immortalized human mast cell lines, particularly LAD2 and HMC-1, allow investigation of MRGPRX2-mediated signaling. LAD2 cells express functional MRGPRX2 and exhibit calcium mobilization, degranulation, and chemokine production following stimulation with SP and other cationic secretagogues [22]. These cells have been instrumental in identifying downstream signaling pathways including PI3K/AKT, PLCγ activation, ERK1/2 phosphorylation, and G-protein-dependent signaling cascades [122].

HMC-1 cells express lower levels of MRGPRX2 and show limited degranulation capacity, although responsiveness can be enhanced experimentally through latrunculin-B pretreatment [96]. Despite their usefulness for mechanistic studies, both cell lines show reduced expression of mast cell proteases such as tryptase and chymase compared with primary mast cells, and HMC-1 cells display an immature phenotype, unsuitable for degranulation assays [95]. Furthermore, variability and lack of methodological standardization across studies using mast cell lines have been reported [123]. These models therefore provide robust information on signaling pathways but have limited physiological relevance.

### 4.2. Primary and Cord Blood-Derived Human Mast Cells

Primary human mast cells provide stronger evidence for the physiological relevance of SP-induced activation. Human skin mast cells have been shown to express high levels of MRGPRX2 and undergo rapid degranulation with histamine and tryptase release following SP stimulation [100]. Studies using freshly isolated mast cells confirmed MRGPRX2-dependent histamine release, demonstrating receptor-specific antagonism [101]. The kinetics of this response are extremely rapid, with histamine release occurring within 10–20 s after stimulation [102], consistent with the rapid onset of neurogenic inflammatory reactions in vivo.

Among the most direct evidence, novel MRGPRX2 antagonists have been shown to inhibit the degranulation of human cord blood-derived mast cells induced by SP and other basic secretagogues, while IgE-challenged cells remained resistant to these antagonists [107]. Supporting evidence showed that SP strongly activated mature human cord blood mast cells, inducing CXCL8 expression and histidine decarboxylase transcription [108]. However, CD133+ cord blood-derived mast cells showed minimal histamine release in response to SP stimulation, suggesting heterogeneity in responsiveness depending on culture protocols [111]. Overall, the evidence strongly supports MRGPRX2-mediated, IgE-independent SP activation in cord blood-derived mast cells.

### 4.3. Murine Mast Cells and Animal Models of Neurogenic Inflammation

In mice, the functional ortholog of the human MRGPRX2 receptor is Mrgprb2, which mediates mast cell activation in response to cationic secretagogues, including SP. The role of this receptor was first demonstrated in knockout models showing that secretagogue-induced histamine release, inflammatory responses, and airway contraction are abolished in Mrgprb2-deficient mice [42]. Subsequent studies confirmed that SP activates murine mast cells through Mrgprb2, inducing rapid intracellular Ca^2+^ mobilization and degranulation with the release of preformed mediators such as histamine, resulting in vasodilation, increased vascular permeability, and local inflammatory responses [113]. More recently, SP-induced degranulation has been demonstrated in Mrgprb2-expressing bone marrow-derived mast cells, further supporting the role of this receptor in murine mast cell activation [55].

These findings have also linked Mrgprb2 signaling to neuroimmune interactions. SP released from sensory neurons contributes to the transmission of pain and itch and can activate cutaneous mast cells through Mrgprb2-dependent mechanisms [114]. However, direct evidence demonstrating that Mrgprb2 signaling itself modulates nociceptor activation remains limited, and most studies support an indirect mast cell–neuron feed-forward loop.

Despite their functional similarity, important differences exist between human MRGPRX2 and its murine ortholog Mrgprb2. While both receptors respond to SP and other cationic peptides and trigger rapid mast cell degranulation through Ca^2+^-dependent signaling pathways, they differ in ligand specificity, pharmacological responsiveness, and expression patterns [55]. Human MRGPRX2 displays broader ligand promiscuity and is highly expressed in connective tissue mast cells, particularly in the skin, whereas murine Mrgprb2 shows a more restricted ligand profile and is primarily studied in bone marrow–derived mast cells and tissue mast cells in experimental models. These interspecies differences represent an important limitation when translating mechanistic findings from murine models to human disease.

Animal models of neurogenic inflammation in both mice and rats have further clarified the roles of Mrgprb2 and NK1 receptors in inflammatory responses. In mice, Mrgprb2 has been shown to mediate inflammatory mechanical and thermal hyperalgesia and to be required for the recruitment of innate immune cells at sites of tissue injury [43]. Similarly, extensive evidence supports a role for NK1 receptor signaling in neurogenic inflammation. In rat models, NK1 receptors have been shown to mediate neutrophil and eosinophil adhesion as well as plasma leakage in tracheal neurogenic inflammation [117], while studies using NK1R-deficient mice demonstrated marked reductions in SP-induced plasma extravasation and leukocyte infiltration [118]. Additional experimental models of neuropathic pain have documented NK1R-associated edema and polymorphonuclear leukocyte accumulation following nerve injury [120]. Collectively, these studies highlight the contribution of both Mrgprb2- and NK1R-dependent pathways to neurogenic inflammatory responses, including edema formation and immune cell recruitment, although direct mechanistic evidence linking these receptors specifically to pain sensitization remains relatively limited.

## 5. Central Nervous System Implications and Stress-Related Pathways

In recent years, increasing attention has been devoted to the role of the SP–mast cell axis within the central nervous system, where neurogenic inflammation is increasingly recognized as a contributor to both neurodegenerative and neuropsychiatric disorders. Mast cells are present in the meninges, perivascular spaces, thalamus, hypothalamus, and other brain regions involved in neuroendocrine regulation and stress responses [124]. In these locations, mast cells are positioned to interact closely with neurons, astrocytes, microglia, and endothelial cells of the BBB. SP released from central or peripheral afferent fibers can activate mast cells in these regions, leading to the release of cytokines, proteases, and vasoactive mediators that may alter BBB permeability and promote neuroinflammation [48,125,126,127,128]. This mechanism has been proposed to facilitate immune cell infiltration into the central nervous system and to sustain glial activation, thereby contributing to chronic neuroinflammatory states [9,15,19,129].

In particular, microglial cells, the resident immune cells of the central nervous system, are also responsive to SP and can be indirectly activated by mast cell-derived mediators. This tri-cellular interaction among neurons, mast cells, and microglia amplifies inflammatory signaling and may exacerbate neuronal dysfunction [130]. In experimental models, SP has been shown to enhance microglial production of pro-inflammatory cytokines and reactive oxygen species, promoting neuronal sensitization and, in some contexts, neurotoxicity. These observations suggest that neurogenic inflammation mediated by SP and mast cells may represent a common pathogenic pathway linking peripheral inflammation to central nervous system disorders [18,20,131,132,133].

Another emerging aspect of SP-mediated neurogenic inflammation involves its interaction with stress-related pathways. Owing to the localization of SP and NK1R in brain regions implicated in the modulation of stress and anxiety, SP is able to mediate cytokine release in response to stressful stimuli, where other neurotransmitters such as dopamine, acetylcholine, serotonin, and noradrenaline are also present. Both psychological and physical stress are known to induce the release of SP from sensory neurons and central stress-responsive circuits. Notably, stress-related factors inhibit the physiological negative feedback mechanism that limits SP release through NK1 receptor activation, resulting in altered SP tissue levels or changes in SP immunoreactivity across multiple brain regions [134].

In cold water-exposed mice, mast cells exhibit increased phagocytic capacity, enhanced adhesion, and elevated free radical production. Additionally, evidence indicates an increased production of cytokines originating both from stressed mast cells and from mast cells activated by SP [96].

Elevated levels of prostaglandin E2 (PGE2) have also been observed in stressed macrophages, which may contribute to stress-induced immune suppression implicated in the exacerbation of inflammatory skin diseases, asthma, irritable bowel syndrome, and chronic pain syndromes. In this context, mast cells act as key transducers of stress signals into inflammatory responses, thereby providing a mechanistic link between psychosocial stressors and disease exacerbations. Furthermore, the ability of SP to modulate hypothalamic–pituitary–adrenal axis activity further highlights its role in integrating neuroendocrine and immune responses [95,96].

These findings suggest potential therapeutic implications for the treatment of depression and anxiety. Both pharmacological and endogenous NK1 receptor antagonists have been shown to attenuate stress-induced mast cell activation. For example, capsaicin, a well-known SP antagonist, has been reported to reduce stress-induced cytokine production in murine model, such as interleukin-6 (IL-6) and tumor necrosis factor-α (TNF-α) by approximately threefold [135,136].

## 6. Pathophysiological Relevance and Peripheral Clinical Correlates of Substance P

**Figure 2:**
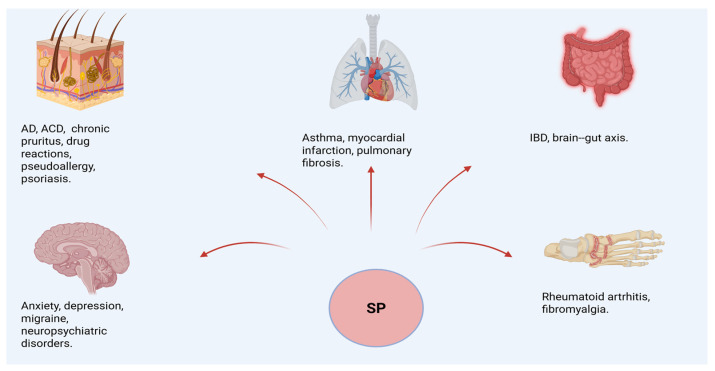
Multisystem clinical spectrum associated with substance P signaling. SP acts as a shared molecular mediator across skin, respiratory and cardiovascular systems, gastrointestinal tract, musculoskeletal tissues, and the central nervous system, contributing to inflammatory, pseudoallergic, pain-related, and neuropsychiatric manifestations. The figure illustrates the broad clinical relevance of SP-associated pathways across multiple organ systems. SP, substance P; AD, atopic dermatitis; ACD, allergic contact dermatitis; IBD, inflammatory bowel disease. Created with BioRender, Ginaldi L. (2026) https://BioRender.com/hkxqywh (accessed on 29 January 2026).

Accumulating evidence from human studies suggests that the SP–MRGPRX2 axis contributes to inflammatory and neuroimmune processes across multiple tissues (Figure 2), although the strength of evidence varies considerably between diseases. Tissue-based studies indicate that MRGPRX2 expression is frequently upregulated in inflammatory conditions affecting the skin, gastrointestinal tract, and airways, even if in many cases the available data remain largely associative and do not clearly distinguish whether SP signaling represents a primary pathogenic driver or a secondary amplification mechanism.

Increased levels of SP and mast cell infiltration have been documented in the synovial fluid of patients with rheumatoid arthritis, in the plasma and skin of individuals with atopic dermatitis, in acute bronchoconstriction and in the bronchoalveolar lavage fluid of patients with asthma [137,138]. SP has also been implicated in histamine-mediated vascular leakage in eczema, and sustained pain and fatigue in fibromyalgia [139,140]. Additional associations have been reported in migraine, myocardial infarction, pulmonary fibrosis, and neuropsychiatric disorders [35,141].

Across these conditions, clinical studies consistently report correlations between elevated SP levels and increased disease severity or adverse outcomes [142,143,144,145,146], although most studies remain correlational and do not establish causality. Rheumatoid arthritis, fibromyalgia, migraine, and neuropsychiatric disorders may represent additional contexts in which SP is associated with symptom severity, pain sensitization, and stress-related exacerbations. In these conditions, the mast cell–SP axis is best interpreted as a network amplifier that links peripheral inflammation, neuronal excitability, and central symptom perception.

Beyond its pro-inflammatory effects, SP influences cellular growth, proliferation, and tissue remodeling in multiple cell types, including fibroblasts, smooth muscle cells, synoviocytes, endothelial cells, and bone marrow-derived cells. These actions contribute to extracellular matrix remodeling, angiogenesis, and wound repair but may also promote pathological fibrosis and chronic inflammation when dysregulated [70,147]. Collectively, the intricate crosstalk between SP and mast cells represents a fundamental mechanism linking the nervous and immune systems [148]. Dysregulation of this axis amplifies inflammatory responses, sustains peripheral and central sensitization, and contributes to the development and persistence of numerous inflammatory, allergic, and pain-related diseases [19,91,149].

Nevertheless, interpretation of clinical findings is further complicated by the heterogeneity of clinical phenotypes and by substantial variability in methods used to quantify SP levels, including differences in biological matrices and analytical techniques. Robust preclinical evidence supports a role for SP in inflammatory skin responses [150], asthma [151], neuropsychiatric conditions such as depression and anxiety [152], and neurodegenerative disorders including Parkinson’s disease [153]. In contrast, clinical evidence remains limited and inconsistent, with many studies providing primarily associative observations.

### Gut, Skin, Airways, and Pseudoallergy: Primary Driver or Secondary Amplifier?

In the gastrointestinal tract, SP participates in visceral hypersensitivity and mucosal immune activation. In irritable bowel syndrome, its role appears to be primarily linked to sensory neuron–mast cell interactions sustaining pain and dysmotility, while in inflammatory bowel disease SP contributes to cytokine production and leukocyte recruitment. In particular, in inflammatory bowel disease, SP promotes interleukin-17 release within the intestinal mucosa, whereas in psoriasis it enhances interleukin-17 production in the skin, contributing to keratinocyte proliferation and chronic inflammation [154,155].

Dermatological disorders provide some of the strongest evidence for a neurogenic component of inflammation. The skin is characterized by dense sensory innervation and a high prevalence of connective tissue mast cells expressing MRGPRX2. In antihistamine-resistant chronic spontaneous urticaria, increased expression of MRGPRX2 and enhanced responsiveness to neuropeptides suggest that SP-driven mast cell activation contributes primarily to disease severity and symptom persistence rather than to disease initiation [156].

Similarly, in the airways SP has been implicated in bronchial hyperreactivity, plasma extravasation, and immune cell recruitment [157]. Experimental models support a mechanistic role for neurogenic inflammation in asthma phenotypes, whereas human studies primarily demonstrate elevated neuropeptide levels and increased mast cell density, consistent with a disease-modifying rather than disease-initiating role [158,159,160].

Taken together, the available evidence suggests that SP-driven mast cell activation more likely acts as a neuroimmune amplifier rather than a primary disease trigger, at least in these models of chronic inflammatory disease, although definitive causal relationships remain difficult to establish from current clinical studies.

However, a different pathogenic scenario may emerge in conditions characterized by transient or limited mast cell activation, such as clinical settings associated with pseudoallergic reactions. As discussed above, the MRGPRX2 receptor displays activation kinetics that differ substantially from the classical IgE–FcεRI pathway, being characterized by rapid, IgE-independent mast cell degranulation triggered by a broad range of cationic ligands. In addition to endogenous neuropeptides such as SP, MRGPRX2 can be activated by several pharmacological agents, including cationic drugs such as fluoroquinolone antibiotics (e.g., ciprofloxacin, levofloxacin), neuromuscular blocking agents, and other basic secretagogues [161].

Certain plant-derived compounds and dietary components have also been reported to interact with mast cell activation pathways, including flavonoids such as quercetin and bioactive molecules present in spices. These latter may contribute to pseudoallergic reactions in susceptible individuals [19].

In these contexts, the cellular and molecular pathways associated with MRGPRX2 activation may translate into specific clinical settings, such as drug-induced acute urticaria, rather than chronic disease forms, as well as early mast cell activation observed in some spice-related adverse reactions [162,163]. In contrast to chronic inflammatory disorders, where the SP–mast cell axis appears to function primarily as an amplification mechanism, these settings may represent situations in which MRGPRX2-mediated mast cell activation acts as a primary driver of the inflammatory response.

Taken together, these observations indicate that both primary pathogenic driver and secondary inflammatory amplifier may operate within SP-mediated mast cell activation. The relative contribution of each mechanism may depend on the temporal dynamics, intensity, and clinical context of mast cell activation.

Further studies in well-characterized clinical cohorts, in addition to experimental models, will be required to clarify the pathophysiological relevance of these mechanisms and their potential translational implications, including the development of targeted therapeutic strategies.

## 7. Therapeutic Implications and Translational Perspectives

The SP–mast cell axis represents an attractive but challenging therapeutic target. NK1R antagonists have been extensively investigated for their potential to modulate pain, inflammation, and mood disorders [70,91,164,165]. While preclinical studies have demonstrated robust anti-inflammatory and analgesic effects, clinical outcomes have been variable, likely reflecting the complexity and redundancy of neuroimmune signaling networks (Table 2). Similarly, direct targeting of mast cell activation through stabilizing agents or inhibition of specific signaling pathways downstream of MRGPRX2 has gained interest. Given the role of MRGPRX2 in pseudo-allergic drug reactions and neurogenic inflammation, selective modulation of this receptor could offer potential therapeutic benefits while avoiding the broad immunosuppressive effects associated with traditional anti-inflammatory drugs [166]. MRGPRX2-targeting DNA aptamer and small molecule antagonists represent potential strategies to target this receptor, as demonstrated by preclinical trials [107,167]. Additional studies have demonstrated that pharmacological inhibition of MRGPRX2 can effectively suppress mast cell activation both in vitro and in ex vivo human skin preparations, supporting its potential therapeutic relevance [168]. Nevertheless, these findings remain confined to preclinical and translational experimental settings, and clinical efficacy in humans has yet to be demonstrated [169].

The discrepancy between robust preclinical results and modest or negative clinical outcomes observed with NK1R antagonists likely reflects the complexity of neuroimmune signaling networks [65]. One major factor is receptor redundancy within the tachykinin system, which allows alternative ligands and receptors to maintain inflammatory signaling despite pharmacological blockade of NK1R.

In addition, SP-induced mast cell activation in human tissues can occur through MRGPRX2, a pathway that is not affected by NK1R antagonism. This receptor bypass mechanism may be particularly relevant in pseudoallergic reactions and neurogenic inflammation of barrier tissues [43,179].

Disease heterogeneity and the lack of patient stratification represent another critical issue [80,180]. NK1R blockade may be effective only in specific endotypes characterized by neurogenic inflammation and SP upregulation, whereas unselected patient populations dilute potential therapeutic signals. Pharmacokinetic limitations and insufficient tissue penetration in highly innervated peripheral compartments may have further contributed to suboptimal clinical responses.

Enzymatic regulation of SP availability represents another potential therapeutic strategy. One possible approach involves the enhancement of neprilysin activity [181]. This endopeptidase plays a key role in the degradation of several neuropeptides, including SP and β-amyloid, thereby contributing, particularly in the brain, to the modulation of inflammatory and neurodegenerative processes [182]. Furthermore, studies in which synthetic SP analogues have been designed and tested suggest a potential future development of peptidic drugs aimed at fine-tuning SP signaling in specific tissues [183]. These analogues were specifically engineered to exhibit reduced biological activity while ensuring strong tissue binding, especially in the brain, by protecting the carboxyl terminus, the site targeted by SP-degrading enzymes [136].

In addition, a well-documented association exists between chronic infections and autoimmune diseases, along with evidence that neurotransmitters can mediate the transition of microglia from an immunosuppressive to a pro-inflammatory state under pathological conditions. On this basis, future combined therapeutic approaches targeting both neuronal and immune components of neurogenic inflammation may be required to achieve sustained clinical efficacy, particularly in chronic inflammatory and pain disorders. A notable example is the observation of reduced insulin resistance and a lower risk of Alzheimer’s disease development in patients with rheumatoid arthritis treated with TNF-α inhibitors [184].

A precision-medicine approach may suggest that therapeutic modulation of the mast cell–SP axis could be more effective in disease endotypes characterized by neurogenic inflammation, mast cell hyperresponsiveness, and stress-related symptom exacerbation.

These conditions may include antihistamine-resistant chronic spontaneous urticaria, chronic pruritus associated with atopic dermatitis, neurogenic asthma phenotypes, irritable bowel syndrome with visceral hypersensitivity, and chronic pain syndromes such as fibromyalgia. In these settings, symptom severity often shows poor correlation with conventional inflammatory biomarkers, supporting the concept of a potential neuroimmune amplification loop.

Identification of such endotypes will require integrated biomarkers combining neuronal, immune, and mast cell-derived mediators.

## 8. Discussion

Neurogenic inflammation represents a key interface between the nervous and immune systems, in which sensory neurons act not only as signal transmitters but also as active effectors of inflammatory responses. Nociceptive afferents can release neuropeptides such as SP and calcitonin gene-related peptide in peripheral tissues in response to chemical, mechanical, or thermal stimuli, triggering vasodilation, increased vascular permeability, and leukocyte recruitment. Although initially considered a protective reflex aimed at neutralizing local insults, neurogenic inflammation is now recognized as a contributor to chronic conditions characterized by persistent neuroimmune activation and tissue dysfunction.

Within this context, mast cells play a central role. As long-lived tissue-resident immune cells located at environmental interfaces and in close proximity to nerves and vessels, they act as sentinels capable of integrating neuronal, immune, and environmental signals. Upon activation, mast cells rapidly release preformed mediators such as histamine, proteases (tryptase, chymase), and heparin and subsequently produce a broad array of lipid mediators, cytokines, and growth factors, thereby influencing vascular responses, immune cell recruitment, barrier function, and tissue remodeling, and fibrosis.

The extension of this model to the CNS also opens new directions. Mast cells at the neurovascular interface may influence BBB integrity and glial activation, linking peripheral neurogenic inflammation to central symptom domains such as fatigue, mood dysregulation, and cognitive complaints.

A tri-cellular axis involving neurons, mast cells, and microglia may amplify inflammatory signaling through cytokines and reactive oxygen species, potentially linking peripheral inflammatory triggers to central symptoms including pain sensitization, fatigue, and mood disturbances.

Clinically, the relevance of the SP–mast cell axis spans a broad spectrum of inflammatory, allergic, and pain-related disorders, involving peripheral neuroimmune responses. Evidence of elevated SP levels and mast cell interaction has been reported in atopic dermatitis, asthma, inflammatory bowel disease, rheumatoid arthritis, and chronic pain syndromes. In many of these conditions, symptom intensity is not fully explained by classical immune markers alone, suggesting that neuroimmune amplification may act through viscero-somatic cross-sensitivity and the convergence-projection phenomena [185,186].

Overall, the convergence of mast cell biology, SP signaling, and neuroimmune feedback loops provides a compelling scenario to reinterpret a range of pathophysiological conditions under the definition of neurogenic inflammation and pseudoallergic mechanisms.

Within the spectrum of mast cell-related diseases such as urticaria, clinical phenomena of pseudoallergy have already been recognized, for example, in adverse reactions to contrast media and non-steroidal anti-inflammatory drugs (NSAIDs), which could not be fully explained by the classical IgE–FcεRI axis and instead suggested mechanisms related to mast cell releasability and drug metabolism [187]. Nevertheless, clinical presentations of acute urticaria independent of IgE-mediated sensitization still occur, particularly among early immediate reactions characterized by poor reproducibility or dose dependence. In these cases, the interaction with the MRGPRX2 receptor may provide a plausible explanation for such pseudoallergic phenomena, as well as for adverse reactions to certain drugs or spices.

Interestingly, chronic urticaria itself may also be functionally influenced by the neurogenic loops discussed above, through mechanisms of secondary neuroimmune amplification that may coexist with other inflammatory foci. Among these, gastrointestinal complaints have been reported as relatively frequent comorbidities in patients with chronic urticaria [21,188].

Within the cutaneous compartment, where MRGPRX2 is highly expressed in mast cells, the implications of neurogenic inflammation may also extend to other dermatological conditions, including atopic dermatitis, allergic contact dermatitis, and pruritic dermatoses, particularly in cases poorly responsive to antihistamines. In such conditions, scratching itself may act as a neurogenic amplifier through stimulation of cutaneous peptidergic nerve endings. This mechanism may be particularly relevant in senile eczema and in forms of secondary non-histaminergic pruritus, possibly related to age-associated anatomical and functional changes, immunosenescence [189,190], and common hepatic or renal comorbidities in elderly individuals [191].

Accordingly, a key strength of this review is the proposal of an integrated SP–mast cell model that links molecular mechanisms of central and peripheral neuroinflammation to multisystem clinical manifestations. By jointly considering NK receptor-dependent and MRGPRX2-mediated signaling and integrating evidence from both in vitro and in vivo studies, this framework highlights mast cells as a shared neuroimmune interface across seemingly distinct diseases, including both Th2-driven allergic conditions and non-Th2 inflammatory disorders. Importantly, this perspective also emphasizes the context and tissue-dependent behavior of the SP–mast cell axis, ranging from a primary inducer of mast cell activation in acute pseudoallergic reactions to a secondary amplifier of neuroimmune signaling in chronic inflammatory states.

From a translational standpoint, however, targeting this axis has proven challenging, and important limitations remain to be addressed.

## 9. Limitations and Future Perspectives

Despite the growing body of literature on the mast cell–SP axis, several limitations should be acknowledged. A substantial proportion of mechanistic data derives from animal models or transformed mast cell lines, whereas studies performed on primary human mast cells remain relatively limited. This imbalance constrains the direct translational interpretation of receptor-specific signaling pathways, while most available clinical studies are associative rather than interventional, and therefore do not allow definitive conclusions regarding causality.

Moreover, the tachykinin system is characterized by marked redundancy, with multiple ligands and receptors capable of partially compensating for each other. This complexity makes it difficult to attribute disease mechanisms to a single neuropeptide pathway and may explain the heterogeneous outcomes observed in clinical trials targeting NK1R.

NK1R antagonists have shown anti-inflammatory and analgesic effects in preclinical models, yet clinical outcomes have been inconsistent. Several factors may explain this discrepancy: the redundancy within the tachykinin system and the presence of multiple receptors; the SP signaling through MRGPRX2 on mast cells, which may bypass NK1R blockade; finally, the disease- and tissue-specific variation in SP production and degradation.

MRGPRX2 itself represents a therapeutic opportunity. On the one hand, its ligand promiscuity and sensitization-independent activation make it an attractive target for pseudoallergic drug reactions and neurogenic inflammatory conditions. Even so, to our knowledge, no human clinical trials targeting MRGPRX2 have been reported to date. Current evidence is limited to preclinical studies, including the development of small-molecule antagonists capable of inhibiting mast cell degranulation and preventing systemic allergic responses in experimental models [178].

In addition, MRGPRX2 is expressed in tissue-resident mast cells across multiple organs, which may complicate tissue-specific therapeutic targeting and raises potential safety concerns related to systemic mast cell modulation and the possibility of off-target effects. These considerations highlight the need for the development of highly selective receptor modulators and for a better understanding of tissue-specific MRGPRX2 signaling.

In addition, reliable biomarkers that identify SP-dependent disease endotypes are currently lacking. Circulating levels of substance P, tissue expression of MRGPRX2, and mast cell mediator signatures are promising candidates, but their clinical validity and reproducibility remain to be established.

Evidence linking SP signaling to human disease spans multiple pathological contexts. For example, cardiovascular studies have reported marked variability in circulating SP concentrations attributable to differences in sampling procedures and analytical methodologies [192], limiting cross-study comparability. Similarly, investigations in neuropsychiatric disorders emphasize that available clinical data remain incomplete and insufficient to define a clear pathogenic role for SP [152,174]. More broadly, many studies rely on small and heterogeneous cohorts and lack standardized protocols for SP quantification or patient stratification. Although gastrointestinal diseases have also been associated with SP signaling, clinical evidence in this area remains comparatively underdeveloped.

Multiple analytical approaches are used to measure SP in neuroinflammation research, with important methodological differences between preclinical and clinical settings. In experimental studies, SP is commonly detected in tissue samples using immunohistochemical techniques [193] and quantified using ELISA-based assays [80], while molecular analyses may employ real-time reverse transcriptase PCR to assess SP mRNA expression in immune cells [194]. In clinical studies, however, measurement of circulating SP presents significant methodological challenges. Reported plasma concentrations can vary considerably depending on sample preparation procedures, such as peptide extraction protocols, and on the analytical detection methods employed [195]. This variability represents a major obstacle to the standardization and comparability of SP measurements across clinical studies.

Furthermore, this review itself has some intrinsic limitations. The available evidence derives from heterogeneous experimental systems and their integration may at times suggest a degree of mechanistic consistency not fully supported across models. In addition, several signaling pathways are presented in an integrative manner, although not all steps have been directly demonstrated in mast cells and are in some cases inferred from other cellular systems. Finally, the hypothesis that certain clinical entities may be reinterpreted within a pseudoallergic framework driven by substance P–mast cell interactions remains largely speculative, as current evidence does not always allow a clear distinction between a primary pathogenic role and a secondary amplifying effect.

Future studies should aim to define biomarker-based strategies for identifying patients with SP-dependent neuroimmune endotypes. In particular, to address the limitations of current studies, spatial transcriptomic mapping of neuroimmune interfaces in barrier tissues and at the neurovascular unit may provide crucial information on the cellular organization of these circuits.

Finally, to improve the design of clinical studies, and considering the heterogeneity of mast cells, tissues, and disease phenotypes, an effective strategy may involve longitudinal clinical studies with careful patient stratification, including the assessment of stress biomarkers and inflammatory markers. Serial measurements of SP and mast cell mediators will also be required to clarify the causal relationships and temporal dynamics underlying neurogenic inflammation.

## 10. Conclusions

The mast cell–SP axis can be interpreted as a modular neuroimmune circuit potentially operating across multiple tissues and coordinating vascular, immune, and neuronal responses. In most chronic inflammatory conditions, available evidence suggests that this axis functions predominantly as a secondary amplifier of ongoing immune responses rather than as a primary etiological trigger. However, in specific contexts such as pseudoallergic reactions or acute mast cell-driven responses, rapid MRGPRX2-mediated activation may instead act as a primary inducer of inflammation. This dual behavior highlights the context- and tissue-dependent nature of SP-mediated mast cell activation.

Despite growing experimental and clinical interest, several important knowledge gaps remain. Mechanistic insights still derive largely from experimental models, whereas studies performed on primary human mast cells and well-characterized clinical cohorts remain limited. In addition, the redundancy of the tachykinin system, the coexistence of NK1R- and MRGPRX2-dependent signaling pathways, and the lack of validated biomarkers capable of identifying SP-dependent disease endotypes continue to represent major obstacles for translational progress.

Future advances will require integrated translational approaches combining experimental models with carefully designed human studies, improved standardization of SP measurement, and biomarker-based patient stratification. A deeper understanding of the mast cell–SP axis may ultimately facilitate the identification of neurogenic inflammatory endotypes and support the development of targeted therapeutic strategies aimed at modulating this neuroimmune interface.

## Figures and Tables

**Table 1:** Experimental evidence supporting substance P–mast cell signaling.

Experimental System	Receptor/Source	Receptor Involved	Functional Outcome
In vitro mast cell lines (LAD2, HMC-1) [95,96,97,98,99]	Human	MRGPRX2, NK1R	Calcium influx, degranulation, cytokine release
Primary human skin mast cells [41,99,100,101,102,103,104,105]	Human	MRGPRX2 (predominant)	Rapid degranulation, histamine and tryptase release
Cord blood–derived mast cells [106,107,108,109,110,111]	Human	MRGPRX2	IgE-independent activation
Murine mast cells [42,55,112,113,114,115]	Mouse (Mrgprb2)	Mrgprb2	Degranulation, vascular permeability, nociceptor activation
Animal models of neurogenic inflammation [19,43,97,116,117,118,119,120,121]	Mouse/rat	Mrgprb2, NK1R	Edema, leukocyte recruitment, pain sensitization

**Table 2:** Clinical and translational evidence supporting the substance P–mast cell axis.

Experimental System	Source	Receptor Involved	Functional Outcome
Human tissue studies (skin, airways, gut, CNS) [19,141,158,159,160,170,171,172]	Human	MRGPRX2 (upregulated expression)	Association with disease activity
Clinical studies measuring SP levels [142,143,144,145,146]	Human	Indirect evidence of SP signaling	Correlation with symptoms and disease severity
Clinical trials with NK1R antagonists [173,174,175,176,177]	Human	NK1R	Variable clinical efficacy
MRGPRX2 modulators [168,169,178,179]	Experimental models (preclinical studies)	MRGPRX2	Inhibition of MC degranulation and attenuation of inflammatory responses in vitro and in vivo. No clinical trials currently available.

CNS: central nervous system; SP: Substance P; MRGPRX2: Mas-Related G Protein–Coupled Receptor X2; NK1R: Neurokinin 1 Receptor; MC: mast cell.

## Data Availability

No new data were created or analyzed in this study. Data sharing is not applicable to this article.

## References

[1] Galli S.J., Tsai M. (2012). IgE and Mast Cells in Allergic Disease. Nat. Med..

[2] Rosenwasser L.J., Boyce J.A. (2003). Mast Cells: Beyond IgE. J. Allergy Clin. Immunol..

[3] Roy S., Ayudhya C.C.N., Thapaliya M., Deepak V., Ali H. (2021). Multifaceted MRGPRX2: New Insight into the Role of Mast Cells in Health and Disease. J. Allergy Clin. Immunol..

[4] Chompunud Na Ayudhya C., Amponnawarat A., Ali H. (2021). Substance P Serves as a Balanced Agonist for MRGPRX2 and a Single Tyrosine Residue Is Required for β-Arrestin Recruitment and Receptor Internalization. Int. J. Mol. Sci..

[5] Schank J.R., Heilig M. (2017). Substance P and the Neurokinin-1 Receptor: The New CRF. Int. Rev. Neurobiol..

[6] Frungieri M.B., Weidinger S., Meineke V., Köhn F.M., Mayerhofer A. (2002). Proliferative Action of Mast-Cell Tryptase Is Mediated by PAR2, COX2, Prostaglandins, and PPARγ: Possible Relevance to Human Fibrotic Disorders. Proc. Natl. Acad. Sci. USA.

[7] van der Kleij H.P., Bienenstock J. (2005). Significance of Conversation between Mast Cells and Nerves. Allergy Asthma Clin. Immunol..

[8] Shaw J., Ramwell P. (1968). Release of a Substance P Polypeptide from the Cerebral Cortex. Am. J. Physiol. Leg. Content.

[9] Nagamine M., Kaitani A., Izawa K., Ando T., Yoshikawa A., Nakamura M., Maehara A., Yamamoto R., Okamoto Y., Wang H. (2024). Neuronal Substance P-Driven MRGPRX2-Dependent Mast Cell Degranulation Products Differentially Promote Vascular Permeability. Front. Immunol..

[10] De Bartolomeis F., Savoia A., Aitella E., Sacerdoti C., Parlato A., Palmieri C., Astarita C. (2014). Urticaria by Neurogenic Switching of Gastroesophageal Chemical-infective Inflammation: A Phenomenon That Should Always Be Evaluated in Suspected Multiple Drug Hypersensitivity. Clin. Transl. Allergy.

[11] Crivellato E., Beltrami C.A., Mallardi F., Ribatti D. (2004). The Mast Cell: An Active Participant or an Innocent Bystander?. Histol. Histopathol..

[12] Héron A., Dubayle D. (2013). A Focus on Mast Cells and Pain. J. Neuroimmunol..

[13] Stachowicz M., Mazurek U., Nowakowska-Zajdel E., Niedworok E., Fatyga E., Muc-Wierzgon M. (2010). Leptin and Its Receptors in Obese Patients with Colorectal Cancer. J. Biol. Regul. Homeost. Agents.

[14] Negrev N., Tashev R., Radev R., Anogeianaki A., Ivanova M. (2011). Hormones of Hypothalamic-Pituitary-Thyroid Axis Are Significant Regulators of Synthesis and Secretion of Vitamin K-Dependent Plasma Coagulation Factors. J. Biol. Regul. Homeost. Agents.

[15] Nicoletti M., Neri G., Maccauro G., Tripodi D., Varvara G., Saggini A., Potalivo G., Castellani M.L., Fulcheri M., Rosati M. (2012). Impact of neuropeptide substance P, an inflammatory compound on arachidonic acid compound generation. Int. J. Immunopathol. Pharmacol..

[16] Guarino F., Cantarella G., Caruso M., Russo C., Mancuso S., Arcidiacono G., Cacciola R.R., Bernardini R., Polosa R. (2011). Endothelial Activation and Injury by Cigarette Smoke Exposure. J. Biol. Regul. Homeost. Agents.

[17] Kokkas A., Goulas A., Stavrianos C., Anogianakis G. (2011). The Role of Cytokines in Pulp Inflammation. J. Biol. Regul. Homeost. Agents.

[18] Zhu J., Qu C., Lu X., Zhang S. (2014). Activation of Microglia by Histamine and Substance P. Cell. Physiol. Biochem..

[19] Thapaliya M., Chompunud Na Ayudhya C., Amponnawarat A., Roy S., Ali H. (2021). Mast Cell-Specific MRGPRX2: A Key Modulator of Neuro-Immune Interaction in Allergic Diseases. Curr. Allergy Asthma Rep..

[20] Atta A.A., Ibrahim W.W., Mohamed A.F., Abdelkader N.F. (2023). Microglia Polarization in Nociplastic Pain: Mechanisms and Perspectives. Inflammopharmacology.

[21] Aitella E., De Bartolomeis F., Savoia A., Fabiani M., Romano M., Astarita C. (2018). The Overlap Syndrome of Urticaria and Gastroesophageal Reflux Disease. PLoS ONE.

[22] Subramanian H., Gupta K., Ali H. (2016). Roles of Mas-Related G Protein–Coupled Receptor X2 on Mast Cell–Mediated Host Defense, Pseudoallergic Drug Reactions, and Chronic Inflammatory Diseases. J. Allergy Clin. Immunol..

[23] Welle M. (1997). Development, Significance, and Heterogeneity of Mast Cells with Particular Regard to the Mast Cell-Specific Proteases Chymase and Tryptase. J. Leukoc. Biol..

[24] Derakhshan T., Boyce J.A., Dwyer D.F. (2022). Defining Mast Cell Differentiation and Heterogeneity through Single-Cell Transcriptomics Analysis. J. Allergy Clin. Immunol..

[25] Cildir G., Pant H., Lopez A.F., Tergaonkar V. (2017). The Transcriptional Program, Functional Heterogeneity, and Clinical Targeting of Mast Cells. J. Exp. Med..

[26] Tauber M., Basso L., Martin J., Bostan L., Pinto M.M., Thierry G.R., Houmadi R., Serhan N., Loste A., Blériot C. (2023). Landscape of Mast Cell Populations across Organs in Mice and Humans. J. Exp. Med..

[27] Valent P., Cerny-Reiterer S., Herrmann H., Mirkina I., George T.I., Sotlar K., Sperr W.R., Horny H.-P. (2010). Phenotypic Heterogeneity, Novel Diagnostic Markers, and Target Expression Profiles in Normal and Neoplastic Human Mast Cells. Best Pract. Res. Clin. Haematol..

[28] Valent P., Schernthaner G.H., Sperr W.R., Fritsch G., Agis H., Willheim M., Bühring H.-J., Orfao A., Escribano L. (2001). Variable Expression of Activation-linked Surface Antigens on Human Mast Cells in Health and Disease. Immunol. Rev..

[29] Frossi B., Mion F., Sibilano R., Danelli L., Pucillo C.E.M. (2018). Is It Time for a New Classification of Mast Cells? What Do We Know about Mast Cell Heterogeneity?. Immunol. Rev..

[30] Parente R., Giudice V., Cardamone C., Serio B., Selleri C., Triggiani M. (2023). Secretory and Membrane-Associated Biomarkers of Mast Cell Activation and Proliferation. Int. J. Mol. Sci..

[31] Akin C., Siebenhaar F., Wechsler J.B., Youngblood B.A., Maurer M. (2024). Detecting Changes in Mast Cell Numbers Versus Activation in Human Disease: A Roadblock for Current Biomarkers?. J. Allergy Clin. Immunol. Pract..

[32] Kawakami T., Galli S.J. (2002). Regulation of Mast-Cell and Basophil Function and Survival by IgE. Nat. Rev. Immunol..

[33] Galli S.J., Gaudenzio N., Tsai M. (2020). Mast Cells in Inflammation and Disease: Recent Progress and Ongoing Concerns. Annu. Rev. Immunol..

[34] Church M.K., Kolkhir P., Metz M., Maurer M. (2018). The Role and Relevance of Mast Cells in Urticaria. Immunol. Rev..

[35] Lao K., Mak H.W.F., Chiang V., Kumar M., Chow B.K.C., Li P.H. (2025). Mas-Related G-Protein Coupled Receptor-X2 and Chemokine (C–C Motif) Ligand 2 Correlate with Disease Activity Among Treatment-Naïve Chinese Patients with Chronic Spontaneous Urticaria. Clin. Exp. Allergy.

[36] Ständer S., Yosipovitch G. (2019). Substance P and Neurokinin 1 Receptor Are New Targets for the Treatment of Chronic Pruritus*. Br. J. Dermatol..

[37] Aitella E., De Martinis M., Romano C., Azzellino G., Ginaldi L. (2025). Neurogenic Inflammation in Allergic Contact Dermatitis. Biomedicines.

[38] Tatemoto K., Nozaki Y., Tsuda R., Konno S., Tomura K., Furuno M., Ogasawara H., Edamura K., Takagi H., Iwamura H. (2006). Immunoglobulin E-Independent Activation of Mast Cell Is Mediated by Mrg Receptors. Biochem. Biophys. Res. Commun..

[39] Kühn H., Kolkhir P., Babina M., Düll M., Frischbutter S., Fok J.S., Jiao Q., Metz M., Scheffel J., Wolf K. (2021). Mas-Related G Protein–Coupled Receptor X2 and Its Activators in Dermatologic Allergies. J. Allergy Clin. Immunol..

[40] Liu Q., Dong X. (2015). The Role of the Mrgpr Receptor Family in Itch. Pharmacology of Itch.

[41] Fujisawa D., Kashiwakura J., Kita H., Kikukawa Y., Fujitani Y., Sasaki-Sakamoto T., Kuroda K., Nunomura S., Hayama K., Terui T. (2014). Expression of Mas-Related Gene X2 on Mast Cells Is Upregulated in the Skin of Patients with Severe Chronic Urticaria. J. Allergy Clin. Immunol..

[42] McNeil B.D., Pundir P., Meeker S., Han L., Undem B.J., Kulka M., Dong X. (2015). Identification of a Mast-Cell-Specific Receptor Crucial for Pseudo-Allergic Drug Reactions. Nature.

[43] Green D.P., Limjunyawong N., Gour N., Pundir P., Dong X. (2019). A Mast-Cell-Specific Receptor Mediates Neurogenic Inflammation and Pain. Neuron.

[44] Zhu Z., Chambers S., Zeng Y., Bhatia M. (2022). Gases in Sepsis: Novel Mediators and Therapeutic Targets. Int. J. Mol. Sci..

[45] Meixiong J., Dong X. (2017). Mas-Related G Protein–Coupled Receptors and the Biology of Itch Sensation. Annu. Rev. Genet..

[46] Wang Z., Franke K., Bal G., Li Z., Zuberbier T., Babina M. (2022). MRGPRX2-Mediated Degranulation of Human Skin Mast Cells Requires the Operation of Gαi, Gαq, Ca++ Channels, ERK1/2 and PI3K—Interconnection between Early and Late Signaling. Cells.

[47] Brown G.C., Neher J.J. (2010). Inflammatory Neurodegeneration and Mechanisms of Microglial Killing of Neurons. Mol. Neurobiol..

[48] Johnson M.B., Young A.D., Marriott I. (2017). The Therapeutic Potential of Targeting Substance P/NK-1R Interactions in Inflammatory CNS Disorders. Front. Cell. Neurosci..

[49] Aitella E., Romano C., Ginaldi L., Cozzolino D. (2025). Mast Cells at the Crossroads of Hypersensitivity Reactions and Neurogenic Inflammation. Int. J. Mol. Sci..

[50] Zhang S., Zeng X., Yang H., Hu G., He S. (2012). Mast Cell Tryptase Induces Microglia Activation via Protease-Activated Receptor 2 Signaling. Cell. Physiol. Biochem..

[51] Marriott I., Bost K.L. Substance P Receptor Mediated Macrophage Responses. Neuroimmune Circuits, Drugs of Abuse, and Infectious Diseases.

[52] Meixiong J., Anderson M., Limjunyawong N., Sabbagh M.F., Hu E., Mack M.R., Oetjen L.K., Wang F., Kim B.S., Dong X. (2019). Activation of Mast-Cell-Expressed Mas-Related G-Protein-Coupled Receptors Drives Non-Histaminergic Itch. Immunity.

[53] Zhu Z., Bhatia M. (2023). Inflammation and Organ Injury the Role of Substance P and Its Receptors. Int. J. Mol. Sci..

[54] Sumpter T., Tkacheva O., Shufesky W., Morelli A., Larregina A. (2014). Signaling via Neurokinin 1 and 2 Receptors Exerts Opposing Effects on Mast Cell Pro-Inflammatory and Th2-Biasing Functions. (HYP3P.347). J. Immunol..

[55] Bawazir M., Roy S., Ali H. (2024). The Development of Murine Bone Marrow-Derived Mast Cells Expressing Functional Human MRGPRX2 for Ex Vivo and in Vivo Studies. Front. Immunol..

[56] Chompunud Na Ayudhya C., Ali H. (2022). Mas-Related G Protein–Coupled Receptor-X2 and Its Role in Non-Immunoglobulin E–Mediated Drug Hypersensitivity. Immunol. Allergy Clin. North Am..

[57] Simmons M.A. (2006). Functional Selectivity of NK1 Receptor Signaling: Peptide Agonists Can Preferentially Produce Receptor Activation or Desensitization. J. Pharmacol. Exp. Ther..

[58] McConalogue K., Corvera C.U., Gamp P.D., Grady E.F., Bunnett N.W. (1998). Desensitization of the Neurokinin-1 Receptor (NK1-R) in Neurons: Effects of Substance P on the Distribution of NK1-R, G_αq/11_, G-Protein Receptor Kinase-2/3, and β-Arrestin-1/2. Mol. Biol. Cell.

[59] Garland A.M., Grady E.F., Lovett M., Vigna S.R., Frucht M.M., Krause J.E., Bunnett N.W. (1996). Mechanisms of Desensitization and Resensitization of G Protein-Coupled Neurokinin1 and Neurokinin2 Receptors. Mol. Pharmacol..

[60] Lazki-Hagenbach P., Kleeblatt E., Ali H., Sagi-Eisenberg R. (2022). Spatiotemporal Patterns of Substance P-Bound MRGPRX2 Reveal a Novel Connection Between Macropinosome Resolution and Secretory Granule Regeneration in Mast Cells. Front. Immunol..

[61] Lazki-Hagenbach P., Kleeblatt E., Fukuda M., Ali H., Sagi-Eisenberg R. (2024). The Underlying Rab Network of MRGPRX2-Stimulated Secretion Unveils the Impact of Receptor Trafficking on Secretory Granule Biogenesis and Secretion. Cells.

[62] Wang Z., Franke K., Zuberbier T., Babina M. (2022). Cytokine Stimulation by MRGPRX2 Occurs with Lower Potency than by FcεRI Aggregation but with Similar Dependence on the Extracellular Signal–Regulated Kinase 1/2 Module in Human Skin Mast Cells. J. Investig. Dermatol..

[63] Carter M., Krause J. (1990). Structure, Expression, and Some Regulatory Mechanisms of the Rat Preprotachykinin Gene Encoding Substance P, Neurokinin A, Neuropeptide K, and Neuropeptide Gamma. J. Neurosci..

[64] Pennefather J.N., Lecci A., Candenas M.L., Patak E., Pinto F.M., Maggi C.A. (2004). Tachykinins and Tachykinin Receptors: A Growing Family. Life Sci..

[65] Steinhoff M.S., von Mentzer B., Geppetti P., Pothoulakis C., Bunnett N.W. (2014). Tachykinins and Their Receptors: Contributions to Physiological Control and the Mechanisms of Disease. Physiol. Rev..

[66] Almeida T.A., Rojo J., Nieto P.M., Pinto F.M., Hernandez M., Martín J.D., Candenas M.L. (2004). Tachykinins and Tachykinin Receptors: Structure and Activity Relationships. Curr. Med. Chem..

[67] Douglas S.D., Leeman S.E. (2011). Neurokinin-1 Receptor: Functional Significance in the Immune System in Reference to Selected Infections and Inflammation. Ann. N. Y. Acad. Sci..

[68] Suvas S. (2017). Role of Substance P Neuropeptide in Inflammation, Wound Healing, and Tissue Homeostasis. J. Immunol..

[69] Mashaghi A., Marmalidou A., Tehrani M., Grace P.M., Pothoulakis C., Dana R. (2016). Neuropeptide Substance P and the Immune Response. Cell. Mol. Life Sci..

[70] Burmeister A.R., Johnson M.B., Chauhan V.S., Moerdyk-Schauwecker M.J., Young A.D., Cooley I.D., Martinez A.N., Ramesh G., Philipp M.T., Marriott I. (2017). Human Microglia and Astrocytes Constitutively Express the Neurokinin-1 Receptor and Functionally Respond to Substance P. J. Neuroinflamm..

[71] Marriott I., Bost K.L. (2001). Expression of Authentic Substance P Receptors in Murine and Human Dendritic Cells. J. Neuroimmunol..

[72] Finkiewicz-Murawiejska L. (1982). Endogenous Peptides of the Central Nervous System: Enkephalins, Endorphins, Substance P. II. Role in the Pain Reaction of the Body. Postep. Hig. Med. Dosw..

[73] Grady E.F., Garland A.M., Gamp P.D., Lovett M., Payan D.G., Bunnett N.W. (1995). Delineation of the Endocytic Pathway of Substance P and Its Seven-Transmembrane Domain NK1 Receptor. Mol. Biol. Cell.

[74] Baker S.J., Morris J.L., Gibbins I.L. (2003). Cloning of a C-Terminally Truncated NK-1 Receptor from Guinea-Pig Nervous System. Mol. Brain Res..

[75] Harris J.A., Faust B., Gondin A.B., Dämgen M.A., Suomivuori C.-M., Veldhuis N.A., Cheng Y., Dror R.O., Thal D.M., Manglik A. (2022). Selective G Protein Signaling Driven by Substance P–Neurokinin Receptor Dynamics. Nat. Chem. Biol..

[76] Saidi M., Kamali S., Beaudry F. (2016). Characterization of Substance P Processing in Mouse Spinal Cord S9 Fractions Using High-Resolution Quadrupole-Orbitrap Mass Spectrometry. Neuropeptides.

[77] Redkiewicz P. (2022). The Regenerative Potential of Substance P. Int. J. Mol. Sci..

[78] Chauhan V.S., Sterka D.G., Gray D.L., Bost K.L., Marriott I. (2008). Neurogenic Exacerbation of Microglial and Astrocyte Responses to *Neisseria meningitidis* and *Borrelia burgdorferi*. J. Immunol..

[79] Rasley A., Bost K.L., Olson J.K., Miller S.D., Marriott I. (2002). Expression of Functional NK-1 Receptors in Murine Microglia. Glia.

[80] Philipp M. (2016). Substance P and Antagonists of the Neurokinin-1 Receptor in Neuroinflammation Associated with Infectious and Neurodegenerative Diseases of the Central Nervous System. J. Neurol. Neuromedicine.

[81] Hang L., Setiawan T., Blum A.M., Urban J., Stoyanoff K., Arihiro S., Reinecker H.-C., Weinstock J.V. (2010). *Heligmosomoides polygyrus* Infection Can Inhibit Colitis through Direct Interaction with Innate Immunity. J. Immunol..

[82] Sierra A., Navascués J., Cuadros M.A., Calvente R., Martín-Oliva D., Ferrer-Martín R.M., Martín-Estebané M., Carrasco M.-C., Marín-Teva J.L. (2014). Expression of Inducible Nitric Oxide Synthase (INOS) in Microglia of the Developing Quail Retina. PLoS ONE.

[83] DeFea K.A., Zalevsky J., Thoma M.S., Déry O., Mullins R.D., Bunnett N.W. (2000). β-Arrestin–Dependent Endocytosis of Proteinase-Activated Receptor 2 Is Required for Intracellular Targeting of Activated Erk1/2. J. Cell Biol..

[84] Zieglgänsberger W. (2019). Substance P and Pain Chronicity. Cell Tissue Res..

[85] Bekhbat M., Rowson S.A., Neigh G.N. (2017). Checks and Balances: The Glucocorticoid Receptor and NFĸB in Good Times and Bad. Front. Neuroendocrinol..

[86] Bradesi S., Svensson C.I., Steinauer J., Pothoulakis C., Yaksh T.L., Mayer E.A. (2009). Role of Spinal Microglia in Visceral Hyperalgesia and NK1R Up-Regulation in a Rat Model of Chronic Stress. Gastroenterology.

[87] Palma C., Minghetti L., Astolfi M., Ambrosini E., Silberstein F.C., Manzini S., Levi G., Aloisi F. (1997). Functional Characterization of Substance P Receptors on Cultured Human Spinal Cord Astrocytes: Synergism of Substance P with Cytokines in Inducing Interleukin-6 and Prostaglandin E2 Production. Glia.

[88] Fitzcharles M.-A., Cohen S.P., Clauw D.J., Littlejohn G., Usui C., Häuser W. (2021). Nociplastic Pain: Towards an Understanding of Prevalent Pain Conditions. Lancet.

[89] Petho G., Reeh P.W. (2012). Sensory and Signaling Mechanisms of Bradykinin, Eicosanoids, Platelet-Activating Factor, and Nitric Oxide in Peripheral Nociceptors. Physiol. Rev..

[90] Veda P. (2011). Why Are Neutrophils Polymorphonuclear?. Eur. J. Inflamm..

[91] Holzer P., Holzer-Petsche U. (1997). Tachykinins in the Gut. Part II. Roles in Neural Excitation, Secretion and Inflammation. Pharmacol. Ther..

[92] Burke N.N., Kerr D.M., Moriarty O., Finn D.P., Roche M. (2014). Minocycline Modulates Neuropathic Pain Behaviour and Cortical M1-M2 Microglial Gene Expression in a Rat Model of Depression. Brain Behav. Immun..

[93] Clauw D.J. (2014). Fibromyalgia: A Clinical Review. JAMA.

[94] Rådinger M., Jensen B.M., Kuehn H.S., Kirshenbaum A., Gilfillan A.M. (2010). Generation, Isolation, and Maintenance of Human Mast Cells and Mast Cell Lines Derived from Peripheral Blood or Cord Blood. Curr. Protoc. Immunol..

[95] Guhl S., Babina M., Neou A., Zuberbier T., Artuc M. (2010). Mast Cell Lines HMC-1 and LAD2 in Comparison with Mature Human Skin Mast Cells—Drastically Reduced Levels of Tryptase and Chymase in Mast Cell Lines. Exp. Dermatol..

[96] Hermans M.A.W., van Stigt A.C., van de Meerendonk S., Schrijver B., van Daele P.L.A., van Hagen P.M., van Splunter M., Dik W.A. (2021). Human Mast Cell Line HMC1 Expresses Functional Mas-Related G-Protein Coupled Receptor 2. Front. Immunol..

[97] Azimi E., Reddy V.B., Shade K.-T.C., Anthony R.M., Talbot S., Pereira P.J.S., Lerner E.A. (2016). Dual Action of Neurokinin-1 Antagonists on Mas-Related GPCRs. JCI Insight.

[98] Manorak W., Idahosa C., Gupta K., Roy S., Panettieri R., Ali H. (2018). Upregulation of Mas-Related G Protein Coupled Receptor X2 in Asthmatic Lung Mast Cells and Its Activation by the Novel Neuropeptide Hemokinin-1. Respir. Res..

[99] Kulka M., Sheen C.H., Tancowny B.P., Grammer L.C., Schleimer R.P. (2008). Neuropeptides Activate Human Mast Cell Degranulation and Chemokine Production. Immunology.

[100] Varricchi G., Pecoraro A., Loffredo S., Poto R., Rivellese F., Genovese A., Marone G., Spadaro G. (2019). Heterogeneity of Human Mast Cells with Respect to MRGPRX2 Receptor Expression and Function. Front. Cell. Neurosci..

[101] Macphee C.H., Dong X., Peng Q., Paone D.V., Skov P.S., Baumann K., Roethke T., Goldspink D.A., Pearson S.K., Wu Z. (2024). Pharmacological Blockade of the Mast Cell MRGPRX2 Receptor Supports Investigation of Its Relevance in Skin Disorders. Front. Immunol..

[102] Lowman M.A., Benyon R.C., Church M.K. (1988). Characterization of Neuropeptide-induced Histamine Release from Human Dispersed Skin Mast Cells. Br. J. Pharmacol..

[103] Foreman J.C. (1987). Substance P and Calcitonin Gene-Related Peptide: Effects on Mast Cells and in Human Skin. Int. Arch. Allergy Immunol..

[104] Ebertz J.M., Hirshman C.A., Kettelkamp N.S., Uno H., Hanifin J.M. (1987). Substance P-Induced Histamine Release in Human Cutaneous Mast Cells. J. Investig. Dermatol..

[105] West P.W., Chéret J., Bahri R., Kiss O., Wu Z., Macphee C.H., Bulfone-Paus S. (2024). The MRGPRX2-Substance P Pathway Regulates Mast Cell Migration. iScience.

[106] Piliponsky A.M., Gleich G.J., Nagler A., Bar I., Levi-Schaffer F. (2003). Non-IgE–Dependent Activation of Human Lung– and Cord Blood–Derived Mast Cells Is Induced by Eosinophil Major Basic Protein and Modulated by the Membrane Form of Stem Cell Factor. Blood.

[107] Ogasawara H., Furuno M., Edamura K., Noguchi M. (2019). Novel MRGPRX2 Antagonists Inhibit IgE-Independent Activation of Human Umbilical Cord Blood-Derived Mast Cells. J. Leukoc. Biol..

[108] Castellani M.L., Ciampoli C., Felaco M., Tetè S., Conti C.M., Salini V., De Amicis D., Orso C., Antinolfi P.L., Caraffa A. (2008). Neuropeptide Substance P Induces MRNA Expression and Secretion of CXCL8 Chemokine, and HDC in Human Umbilical Cord Blood Mast Cells. Clin. Investig. Med..

[109] Iikura M., Suto H., Kajiwara N., Oboki K., Ohno T., Okayama Y., Saito H., Galli S.J., Nakae S. (2007). IL-33 Can Promote Survival, Adhesion and Cytokine Production in Human Mast Cells. Lab. Investig..

[110] Theoharides T.C., Zhang B., Kempuraj D., Tagen M., Vasiadi M., Angelidou A., Alysandratos K.-D., Kalogeromitros D., Asadi S., Stavrianeas N. (2010). IL-33 Augments Substance P–Induced VEGF Secretion from Human Mast Cells and Is Increased in Psoriatic Skin. Proc. Natl. Acad. Sci. USA.

[111] Jensen B.M., Frandsen P.M., Raaby E.M., Schiøtz P.O., Skov P.S., Poulsen L.K. (2014). Molecular and Stimulus-Response Profiles Illustrate Heterogeneity between Peripheral and Cord Blood-Derived Human Mast Cells. J. Leukoc. Biol..

[112] Ansel J.C., Brown J.R., Payan D.G., Brown M.A. (1993). Substance P Selectively Activates TNF-Alpha Gene Expression in Murine Mast Cells. J. Immunol..

[113] Grimbaldeston M.A. (2015). Mast Cell-MrgprB2: Sensing Secretagogues or a Means to Overreact?. Immunol. Cell Biol..

[114] Quan P.L., Sabaté-Brescó M., Guo Y., Martín M., Gastaminza G. (2021). The Multifaceted Mas-Related G Protein-Coupled Receptor Member X2 in Allergic Diseases and Beyond. Int. J. Mol. Sci..

[115] Sutradhar S., Ali H. (2024). Mast Cell MrgprB2 in Neuroimmune Interaction in IgE-Mediated Airway Inflammation and Its Modulation by β-Arrestin2. Front. Immunol..

[116] Suzuki R., Furuno T., McKay D.M., Wolvers D., Teshima R., Nakanishi M., Bienenstock J. (1999). Direct Neurite-Mast Cell Communication In Vitro Occurs Via the Neuropeptide Substance P. J. Immunol..

[117] Baluk P., Bertrand C., Geppetti P., McDonald D.M., Nadel J.A. (1995). NK1 Receptors Mediate Leukocyte Adhesion in Neurogenic Inflammation in the Rat Trachea. Am. J. Physiol. -Lung Cell. Mol. Physiol..

[118] Grady E.F., Yoshimi S.K., Maa J., Valeroso D., Vartanian R.K., Rahim S., Kim E.H., Gerard C., Gerard N., Bunnett N.W. (2000). Substance P Mediates Inflammatory Oedema in Acute Pancreatitis via Activation of the Neurokinin-1 Receptor in Rats and Mice. Br. J. Pharmacol..

[119] McDonald D.M., Bowden J.J., Baluk P., Bunnett N.W. (1996). Neurogenic Inflammation: A model for studying efferent actions of sensory nerves. Adv. Exp. Med. Biol..

[120] Daemen M., Kurvers H., Kitslaar P., Slaaf D., Bullens P., Van den Wildenberg F. (1998). Neurogenic Inf Ammation in an Animal Model of Neuropathic Pain. Neurol. Res..

[121] Rittner H.L., Lux C., Labuz D., Mousa S.A., Schäfer M., Stein C., Brack A. (2007). Neurokinin-1 Receptor Antagonists Inhibit the Recruitment of Opioid-Containing Leukocytes and Impair Peripheral Antinociception. Anesthesiology.

[122] Zhang F., Hong F., Wang L., Fu R., Qi J., Yu B. (2022). MrgprX2 Regulates Mast Cell Degranulation through PI3K/AKT and PLCγ Signaling in Pseudo-Allergic Reactions. Int. Immunopharmacol..

[123] Yip A.J.W., Lee Y.Z., Kow A.S.F., Wong C.S.-A., Lee M.-T., Tham C.L., Tan J.W. (2025). Current Utilization Trend of Immortalized Mast Cell Lines in Allergy Research: A Systematic Review. Immunol. Res..

[124] Ikarashi Y., Yuzurihara M. (2002). Experimental Anxiety Induced by Histaminergics in Mast Cell-Deficient and Congenitally Normal Mice. Pharmacol. Biochem. Behav..

[125] Gerling I.J. (1989). Interaction of Stimulus Parameters on the Auditory Brain Stem Response: A Normal Variant. Ear Hear..

[126] Dolev E. (1987). A Gland in a Search of a Function: The Parathyroid Glands and the Explanations of Tetany 1903–1926. J. Hist. Med. Allied Sci..

[127] Hickey W.F. (1999). Leukocyte Traffic in the Central Nervous System: The Participants and Their Roles. Semin. Immunol..

[128] Lossinsky A.S., Shivers R.R. (2004). Structural Pathways for Macromolecular and Cellular Transport across the Blood-Brain Barrier during Inflammatory Conditions. Review. Histol. Histopathol..

[129] Rigante D., Zampetti A., Bersani G., Candelli M., Piras A., Rendeli C., Antuzzi D., Feliciani C., Stabile A. (2011). Serum Interleukin-18 in Children with Henoch-Schönlein Purpura: A Promising Marker of Disease Activity?. Eur. J. Inflamm..

[130] Netea M.G., Balkwill F., Chonchol M., Cominelli F., Donath M.Y., Giamarellos-Bourboulis E.J., Golenbock D., Gresnigt M.S., Heneka M.T., Hoffman H.M. (2017). A Guiding Map for Inflammation. Nat. Immunol..

[131] Tawfik V.L., Nutile-McMenemy N., Lacroix-Fralish M.L., Deleo J.A. (2007). Efficacy of Propentofylline, a Glial Modulating Agent, on Existing Mechanical Allodynia Following Peripheral Nerve Injury. Brain Behav. Immun..

[132] Kiguchi N., Kobayashi Y., Saika F., Sakaguchi H., Maeda T., Kishioka S. (2015). Peripheral Interleukin-4 Ameliorates Inflammatory Macrophage-Dependent Neuropathic Pain. Pain.

[133] Maggi C.A. (2000). Principles of Tachykininergic Co-Transmission in the Peripheral and Enteric Nervous System. Regul. Pept..

[134] Ebner K., Singewald N. (2006). The Role of Substance P in Stress and Anxiety Responses. Amino Acids.

[135] Chancellor-Freeland C., Zhu G.F., Kage R., Beller D.I., Leeman S.E., Black P.H. (1995). Substance P and Stress-Induced Changes in Macrophages. Ann. N. Y. Acad. Sci..

[136] Sandberg B.E.B., Lee C., Hanley M.R., Iversen L.L. (1981). Synthesis and Biological Properties of Enzyme-Resistant Analogues of Substance P. Eur. J. Biochem..

[137] Dahamsheh Z., Bellomo R.G., Saggini R., Barassi G., Saggini A. (2011). The Prevalence of Rheumatoid Arthritis in the South of Jordan. Eur. J. Inflamm..

[138] Jessell T.M., Iversen L.L. (1977). Inhibition of Substance P Release from the Isolated Rat Substantia Nigra by GABA [Proceedings]. Br. J. Pharmacol..

[139] Puneet P., Hegde A., Ng S.W., Lau H.Y., Lu J., Moochhala S.M., Bhatia M. (2006). Preprotachykinin-A Gene Products Are Key Mediators of Lung Injury in Polymicrobial Sepsis. J. Immunol..

[140] Binici D.N., Güneş N., Kayataş K., Pişkinpaşa N. (2011). The Effects of Interferon-A2b on Intestinal Flora in Peritoneal Fibrosis. Eur. J. Inflamm..

[141] Coccaro E.F., Lee R., Owens M.J., Kinkead B., Nemeroff C.B. (2012). Cerebrospinal Fluid Substance P-Like Immunoreactivity Correlates with Aggression in Personality Disordered Subjects. Biol. Psychiatry.

[142] Smieszek S. (2021). Late Breaking Abstract—Increased Substance P Levels in COVID-19 Hospitalized Patients. Proceedings of the Airway Cell Biology and Immunopathology.

[143] Robinson P., Okhuysen P.C., Chappell C.L., Weinstock J.V., Lewis D.E., Actor J.K., White A.C. (2003). Substance P Expression Correlates with Severity of Diarrhea in Cryptosporidiosis. J. Infect. Dis..

[144] Barbosa-Cobos R.E., Lugo-Zamudio G., Flores-Estrada J., Becerril-Mendoza L.T., Rodríguez-Henríquez P., Torres-González R., Moreno-Eutimio M.A., Ramirez-Bello J., Moreno J. (2018). Serum Substance P: An Indicator of Disease Activity and Subclinical Inflammation in Rheumatoid Arthritis. Clin. Rheumatol..

[145] Lorente L., Martín M.M., Almeida T., Hernández M., Ramos L., Argueso M., Cáceres J.J., Solé-Violán J., Jiménez A. (2015). Serum Substance P Levels Are Associated with Severity and Mortality in Patients with Severe Traumatic Brain Injury. Crit. Care.

[146] Russell I.J., Orr M.D., Littman B., Vipraio G.A., Alboukrek D., Michalek J.E., Lopez Y., Mackillip F. (1994). Elevated Cerebrospinal Fluid Levels of Substance p in Patients with the Fibromyalgia Syndrome. Arthritis Rheum..

[147] Oehme P., Hecht K., Faulhaber H.D., Nieber K., Roske I., Rathsack R. (1987). Relationship of Substance P to Catecholamines, Stress, and Hypertension. J. Cardiovasc. Pharmacol..

[148] Jucá P.M., de Almeida Duque É., Covre L.H.H., Mariano K.A.A., Munhoz C.D. (2024). Microglia and systemic immunity. Adv. Neurobiol..

[149] Kabata H., Artis D. (2019). Neuro-Immune Crosstalk and Allergic Inflammation. J. Clin. Investig..

[150] Matis W.L., Lavker R.M., Murphy G.F. (1990). Substance P Induces the Expression of an Endothelial-Leukocyte Adhesion Molecule by Microvascular Endothelium. J. Investig. Dermatol..

[151] Todo-Bom A., Mota Pinto A., Vale Pereira S., Dourado M., Alves V., Santos Rosa M. (2006). Substance P in Long-Lasting Asthma. Allergy Clin. Immunol. Int. J. World Allergy Organ..

[152] Herpfer I., Lieb K. (2003). Substance P and Substance P Receptor Antagonists in the Pathogenesis and Treatment of Affective Disorders. World J. Biol. Psychiatry.

[153] Tirassa P., Schirinzi T., Raspa M., Ralli M., Greco A., Polimeni A., Possenti R., Mercuri N.B., Severini C. (2021). What Substance P Might Tell Us about the Prognosis and Mechanism of Parkinson’s Disease?. Neurosci. Biobehav. Rev..

[154] Krivoy W., Kroeger D. (1963). The Neurogenic Activity of High Potency Substance P. Experientia.

[155] Katayama Y., North R.A. (1978). Does Substance P Mediate Slow Synaptic Excitation within the Myenteric Plexus?. Nature.

[156] Marek-Jozefowicz L., Nedoszytko B., Grochocka M., Żmijewski M.A., Czajkowski R., Cubała W.J., Slominski A.T. (2023). Molecular Mechanisms of Neurogenic Inflammation of the Skin. Int. J. Mol. Sci..

[157] Baluk P. (1997). Neurogenic Inflammation in Skin and Airways. J. Investig. Dermatol. Symp. Proc..

[158] Groneberg D.A., Quarcoo D., Frossard N., Fischer A. (2004). Neurogenic Mechanisms in Bronchial Inflammatory Diseases. Allergy.

[159] Barnes P.J. (2001). Neurogenic Inflammation in the Airways. Respir. Physiol..

[160] Claire A.B., Liam G. (2007). Heaney Neurogenic Inflammation and Asthma. Inflamm. Allergy-Drug Targets.

[161] McNeil B.D. (2021). MRGPRX2 and Adverse Drug Reactions. Front. Immunol..

[162] Lin X.P., Magnusson J., Ahlstedt S., Dahlman-Höglund A., Hanson L.Å., Magnusson O., Bengtsson U., Telemo E. (2002). Local Allergic Reaction in Food-Hypersensitive Adults despite a Lack of Systemic Food-Specific IgE. J. Allergy Clin. Immunol..

[163] Bradatan E., Sabouraud D. (2020). Spice Reactions in Children: Allergic or Not? Cases Reports and Literature Review. World Allergy Organ. J..

[164] Visser H.K. (1977). Rising Incidence of Surgical Treatment of Cryptorchism (Orchidopexy) in Our Country. Ned. Tijdschr. Geneeskd..

[165] Martinez A.N., Ramesh G., Jacobs M.B., Philipp M.T. (2015). Antagonist of the Neurokinin-1 Receptor Curbs Neuroinflammation in Ex Vivo and in Vitro Models of Lyme Neuroborreliosis. J. Neuroinflamm..

[166] Vanegas H., Schaible H.-G. (2001). Prostaglandins and Cycloxygenases in the Spinal Cord. Prog. Neurobiol..

[167] Suzuki Y., Liu S., Ogasawara T., Sawasaki T., Takasaki Y., Yorozuya T., Mogi M. (2020). A Novel MRGPRX2-Targeting Antagonistic DNA Aptamer Inhibits Histamine Release and Prevents Mast Cell-Mediated Anaphylaxis. Eur. J. Pharmacol..

[168] Wollam J., Solomon M., Villescaz C., Lanier M., Evans S., Bacon C., Freeman D., Vasquez A., Vest A., Napora J. (2024). Inhibition of Mast Cell Degranulation by Novel Small Molecule MRGPRX2 Antagonists. J. Allergy Clin. Immunol..

[169] Castells M., Madden M., Oskeritzian C.A. (2025). Mast Cells and Mas-Related G Protein-Coupled Receptor X2: Itching for Novel Pathophysiological Insights to Clinical Relevance. Curr. Allergy Asthma Rep..

[170] Pyatilova P., Ashry T., Luo Y., He J., Bonnekoh H., Jiao Q., Moñino-Romero S., Hu M., Scheffel J., Frischbutter S. (2022). The Number of MRGPRX2-Expressing Cells Is Increased in Skin Lesions of Patients with Indolent Systemic Mastocytosis, but Is Not Linked to Symptom Severity. Front. Immunol..

[171] Chen E., Chuang L., Giri M., Villaverde N., Hsu N., Sabic K., Joshowitz S., Gettler K., Nayar S., Chai Z. (2021). Inflamed Ulcerative Colitis Regions Associated with MRGPRX2-Mediated Mast Cell Degranulation and Cell Activation Modules, Defining a New Therapeutic Target. Gastroenterology.

[172] Glover S.C., Williams H., Pride Y., Owings A.H., Robinson T., Lyons J., Deepak V., Ali H. (2022). Increased Tryptase Expression in HαT Is Associated with Upregulation of Epithelia-Derived MRGPRX2 Agonists and MRGPRX2+ MCs in the GI Mucosa. J. Immunol..

[173] Rupniak N.M.J., Kramer M.S. (2017). NK1 Receptor Antagonists for Depression: Why a Validated Concept Was Abandoned. J. Affect. Disord..

[174] Herpfer I., Lieb K. (2005). Substance P Receptor Antagonists in Psychiatry. CNS Drugs.

[175] Hafizi S., Chandra P., Cowen P.J. (2007). Neurokinin-1 Receptor Antagonists as Novel Antidepressants: Trials and Tribulations. Br. J. Psychiatry.

[176] Kramer M.S. (2000). Update on Substance P (NK-1 Receptor) Antagonists in Clinical Trials for Depression. Neuropeptides.

[177] Hill R. (2000). NK1 (Substance P) Receptor Antagonists—Why Are They Not Analgesic in Humans?. Trends Pharmacol. Sci..

[178] Kumar M., Duraisamy K., Annapureddy R.R., Chan C.B., Chow B.K.C. (2023). Novel Small Molecule MRGPRX2 Antagonists Inhibit a Murine Model of Allergic Reaction. J. Allergy Clin. Immunol..

[179] Ali H. (2016). Mas-Related G Protein Coupled Receptor-X2: A Potential New Target for Modulating Mast Cell-Mediated Allergic and Inflammatory Diseases. J. Immunobiol..

[180] O’Connor T.M., O’Connell J., O’Brien D.I., Goode T., Bredin C.P., Shanahan F. (2004). The Role of Substance P in Inflammatory Disease. J. Cell. Physiol..

[181] Sekizawa K., Xia Jia Y., Ebihara T., Hirose Y., Hirayama Y., Sasaki H. (1996). Role of Substance P in Cough. Pulm. Pharmacol..

[182] Saxena S.K., Ansari S., Maurya V.K., Kumar S., Sharma D., Malhotra H.S., Tiwari S., Srivastava C., Paweska J.T., Abdel-Moneim A.S. (2024). Neprilysin-Mediated Amyloid Beta Clearance and Its Therapeutic Implications in Neurodegenerative Disorders. ACS Pharmacol. Transl. Sci..

[183] Kruszyński M., Kupryszewski G., Misterek K., Gumułka S. (1990). Synthesis and Some Biological Properties of the Hexapeptide Analog of Substance P with a C-Terminal Thioamide Group. Pol. J. Pharmacol. Pharm..

[184] Furman D., Campisi J., Verdin E., Carrera-Bastos P., Targ S., Franceschi C., Ferrucci L., Gilroy D.W., Fasano A., Miller G.W. (2019). Chronic Inflammation in the Etiology of Disease across the Life Span. Nat. Med..

[185] Jin Q., Chang Y., Lu C., Chen L., Wang Y. (2023). Referred Pain: Characteristics, Possible Mechanisms, and Clinical Management. Front. Neurol..

[186] Heatley M., Rose K., Weston C. (2005). The Heart and the Oesophagus: Intimate Relations. Postgrad. Med. J..

[187] Vitte J., Vibhushan S., Bratti M., Montero-Hernandez J.E., Blank U. (2022). Allergy, Anaphylaxis, and Nonallergic Hypersensitivity: IgE, Mast Cells, and Beyond. Med. Princ. Pract..

[188] Abadeh A., Herman S.M., Abdalian R. (2023). The Prevalence of Gastrointestinal Symptoms and Cobalamin Deficiency in Patients with Chronic Urticaria. Allergy Asthma Clin. Immunol..

[189] Aitella E., Azzellino G., Cammisuli B.A., De Benedictis C., Di Mattia D., Romano C., Ginaldi L., De Martinis M. (2026). Immunosenescence and Allergy: Molecular and Cellular Links Between Inflammaging, Neuro-Immune Aging, and Response to Biologic Therapies. Int. J. Mol. Sci..

[190] De Martinis M., Franceschi C., Monti D., Ginaldi L. (2007). Apoptosis Remodeling in Immunosenescence: Implications for Strategies to Delay Ageing. Curr. Med. Chem..

[191] Monti M., Caruso T., Castellaccio A., De Giorgi I., Cavallini G., Manca M.L., Lorini S., Marri S., Petraccia L., Madia F. (2025). HBV and HCV Testing Outcomes among Marginalized Communities in Italy, 2019–2024: A Prospective Study. Lancet Reg. Health Eur..

[192] Feickert M., Burckhardt B.B. (2019). Substance P in Cardiovascular Diseases—A Bioanalytical Review. Clin. Chim. Acta.

[193] Tuncer L., Alacam T., Oral B. (2004). Substance P Expression Is Elevated in Inflamed Human Periradicular Tissue. J. Endod..

[194] Lai J.-P., Lai S., Tuluc F., Tansky M.F., Kilpatrick L.E., Leeman S.E., Douglas S.D. (2008). Differences in the Length of the Carboxyl Terminus Mediate Functional Properties of Neurokinin-1 Receptor. Proc. Natl. Acad. Sci. USA.

[195] Campbell D.E., Raftery N., Tustin R., Tustin N.B., DeSilvio M.L., Cnaan A., Aye P.P., Lackner A.A., Douglas S.D. (2006). Measurement of Plasma-Derived Substance P: Biological, Methodological, and Statistical Considerations. Clin. Vaccine Immunol..

